# Clinical effects of a selective urate reabsorption inhibitor dotinurad in patients with hyperuricemia and treated hypertension: a multicenter, prospective, exploratory study (DIANA)

**DOI:** 10.1186/s40001-023-01208-1

**Published:** 2023-07-17

**Authors:** Atsushi Tanaka, Isao Taguchi, Itaru Hisauchi, Hisako Yoshida, Michio Shimabukuro, Hiroshi Hongo, Tetsuya Ishikawa, Toshiaki Kadokami, Shusuke Yagi, Masataka Sata, Koichi Node, the DIANA study investigators, Machiko Asaka, Kohei Kamishita, Tetsuya Kaneko, Kohei Kaneta, Masahiro Natsuaki, Aya Shiraki, Shinjo Sonoda, Motoko Tago, Ayumu Yajima, Kensuke Yokoi, Goro Yoshioka, Ryo Nakamura, Junichiro Nishi, Ken Onizuka, Takayuki Ise, Muneyuki Kadota, Yutaka Kawabata, Kenya Kusunose, Kazuhisa Matsumoto, Tomomi Matsuura, Yuichiro Okushi, Hiromitsu Seno, Takeshi Soeki, Kumiko Suto, Tomonori Takahashi, Takeshi Tobiume, Tetsuzo Wakatsuki, Hirotsugu Yamada, Koji Yamaguchi, Yuki Hotta, Mariko Iwasaki, Junichiro Kazama, Yu Saito, Masahiro Sato, Yoshinori Takiguchi, Hayato Tanabe, Kiriko Watanabe, Mizuki Yamaguchi, Sachiko Tomita, Mikiko Kagiyama, Keiko Onodera

**Affiliations:** 1 Department of Cardiovascular Medicine, Saga University, 5-5-1 Nabeshima, Saga, 849-8501 Japan; 2 Department of Cardiology, Dokkyo Medical University Saitama Medical Center, Saitama, Japan; 3 Department of Medical Statistics, Osaka Metropolitan University, Osaka, Japan; 4 Department of Diabetes, Endocrinology, and Metabolism, Fukushima Medical University School of Medicine, Fukushima, Japan; 5 Cardiovascular Medicine, Fukuoka Saiseikai Futsukaichi Hospital, Chikushino, Japan; 6 Department of Cardiovascular Medicine, Tokushima University Hospital, Tokushima, Japan

**Keywords:** Selective urate transporter 1 inhibitor, Dotinurad, Hyperuricemia, Arterial stiffness, Oxidative stress

## Abstract

**Introduction:**

Dotinurad is a newer urate-lowering agent that selectively inhibits urate transporter 1 in the renal proximal tubule and increases urinary urate excretion. Currently, little is known about the clinical efficacies of dotinurad in patients with hyperuricemia and hypertension. The aim of this study was to assess the clinical effects of a selective urate reabsorption inhibitor dotinurad on serum uric acid (SUA) levels and relevant vascular markers in patients with hyperuricemia and treated hypertension.

**Methods:**

This investigator-initiated, multicenter, prospective, single-arm, open-label, exploratory clinical trial in Japan enrolled patients with hyperuricemia and treated hypertension who received a 24-week dotinurad therapy (a starting dose at 0.5 mg once daily and up-titrated to 2 mg once daily). The primary endpoint was a percentage change in the SUA level from baseline to week 24. The secondary endpoints were cardiovascular and metabolic measurements, including changes in the cardio-ankle vascular index (CAVI) and derivatives of reactive oxygen metabolites (d-ROMs) concentration at week 24.

**Results:**

Fifty patients (mean age 70.5 ± 11.0 years, with 76.0% being men, and mean SUA level 8.5 ± 1.2 mg/dL) were included in the analysis. The percentage change from baseline in the SUA level at week 24 was − 35.8% (95% confidence interval [CI] − 39.7% to − 32.0%, *P* < 0.001), with approximately three quarters of patients achieving an SUA level of ≤ 6.0 mg/dL at week 24. The proportional changes from baseline in the geometric mean of CAVI and d-ROMs at week 24 were 0.96 (95% CI 0.92 to 1.00, *P* = 0.044) and 0.96 (95% CI 0.92 to 1.00, *P* = 0.044), respectively.

**Conclusion:**

In addition to meaningful SUA-lowering effects, 24 weeks of dotinurad therapy may favorably affect arterial stiffness and oxidative stress markers, suggesting off-target vascular protection of dotinurad. Further research is expected to verify our findings and elucidate the entire off-target effects of dotinurad.

*Trial registration* jRCTs021210013, registration date June 24, 2021

**Supplementary Information:**

The online version contains supplementary material available at 10.1186/s40001-023-01208-1.

## Introduction

Hyperuricemia is a residual risk factor or marker of cardiovascular disease (CVD) [1]. Specifically, patients with hyperuricemia frequently experience hypertension [2, 3], which synergistically facilitates the development of morbidities such as gout and even CVD. Therefore, optimal preventive measures are needed for that condition. However, whether conventional uric acid-lowering medications, such as xanthine oxidase (XO) inhibitors [4, 5], can provide cardiovascular benefits regardless of urate-lowering effect, is still controversial.

Dotinurad is a newer urate-lowering agent that suppresses uric acid reabsorption through the selective inhibition of urate transporter 1 (URAT1) in the proximal renal tubules [6], and it was first approved in Japan in 2020 for the treatment of hyperuricemia, irrespective of gout. Patients with metabolic syndrome, including hypertension, have increased uric acid reabsorption through URAT1 activation [7], resulting in increased serum uric acid (SUA) levels. Moreover, inflammation and excess oxidative stress induced when uric acid is taken up into cells via URAT1 expressed on vascular smooth muscle and vascular endothelial cells have been suggested to be involved in the development of atherosclerosis and subsequent CVD [8, 9]. These suggest that URAT1 inhibition is clinically suitable for patients with hyperuricemia at the risk of CVD and is even effective for mitigating CVD risk. However, the clinical effectiveness of dotinurad therapy in the real-world setting is still unknown. Thus, in this study, we sought to investigate the effect of dotinurad therapy on lowering urate levels and relevant cardiometabolic measures in patients with hyperuricemia and treated hypertension.

## Methods

### Study design and population

This investigator-initiated, multicenter, prospective, open-label, single-arm, exploratory clinical trial (effect of dotinurad in hyperuricemia with hypertension [DIANA]; jRCTs021210013) was conducted to assess the net clinical effects of dotinurad therapy on SUA levels and vascular function in Japanese patients with hyperuricemia (SUA > 7.0 mg/dL) without active gouty arthritis and had treated hypertension. Detailed inclusion and exclusion criteria are provided in Additional file 1: Table S1.

After written informed consent acquisition and eligibility assessment, eligible patients received dotinurad (starting dose at 0.5 mg once daily and up-titrated to a maintenance 2 mg once daily) for 24 weeks. The dose of dotinurad was requested to be up-titrated, in principle, to 1.0 mg once daily at week 4 and 2 mg once daily at week 8. If the SUA level exceeds 6.0 mg/dL in spite of maintenance dose of dotinurad, a further escalation of dotinurad to a maximum of 4 mg once daily was allowed. If a patient had been receiving any uric acid-lowering drug at the time of consent, dotinurad therapy was initiated after at least a 27-day break in the prior drug. The follow-up visits were made at weeks 4, 8, 12, and 24 after the initiation of dotinurad therapy. The background therapy of each patient remained unchanged during the study interval, and the use of any urate-lowering agent other than dotinurad was prohibited.

This study was approved by the Fukushima Medical University Certified Review Board (No. F2021002 on June 10, 2021) and conducted in accordance with the Declaration of Helsinki and the Clinical Trial Act in Japan.

### Study endpoints

The primary endpoint was a percentage change in the SUA level from baseline to week 24 of dotinurad therapy. The secondary endpoints included relevant clinical measurements, such as changes in the SUA level at weeks 4, 8, 12, and 24, proportions of patients who achieved an SUA level of ≤ 6.0 mg/dL at corresponding weeks, change in blood pressure (BP) over 24 weeks, and changes in the cardio-ankle vascular index (CAVI) and several biomarkers including C-reactive protein (CRP, mg/dL), interleukin-6 (IL-6, pg/mL), growth differentiation factor 15 (GDF-15, pg/mL), N-terminal pro-brain natriuretic peptide (NT-proBNP, pg/mL), high-sensitivity troponin T (hs-TnT, pg/mL), derivatives of reactive oxygen metabolites (d-ROMs, U.CARR), and urinary albumin creatinine ratio (UACR) at week 24. CRP, IL-6, GFF-15, NT-proBNP, and hs-TnT levels were measured at a central commercial-based laboratory (SRL, Inc., Tokyo, Japan). The evaluation of d-ROMs concentrations was centrally conducted at an academic laboratory (Department of Cardiology, Dokkyo Medical University Saitama Medical Center, Saitama, Japan) by measuring hydroperoxide serum levels (Diacron, Grosseto, Italy) and quantified by a photometer (FREE, Diacron) at a wavelength of 505 nm [10]. UACR was measured from the urine sample obtained at the time of the office visit. The estimated glomerular filtration rate (eGFR), estimated by a revised equations from serum creatinine for Japanese [11], and Fib-4 index were also measured at baseline and weeks 12 and 24.

### CAVI measurement

Detailed methods for measuring CAVI were described previously [12]. Briefly, using a VeSera device (Fukuda Denshi, Tokyo, Japan), the CAVI was automatically measured based on the standard protocol on the right and left sides of the body, and the mean value of each one was used in the analysis.

### Statistics

The target sample size of the DIANA study was initially based on the percent change in the SUA level (effect size) at 14 weeks in the phase 3 study of dotinurad and at 34 weeks in the long-term study, which ranged from 41.82% to 46.73% [13–15]. Then, in this study, the percent change in the SUA level at 24 weeks was assumed to be 42% (standard deviation 12%). If the lower limit of its 95% confidence interval (CI) was > 30%, we considered it an effective urate-lowering effect. As regards the primary endpoint, the number of cases required would be < 10 when calculated with *α* = 0.05 and 1 − β (power) = 0.80. However, this exploratory study also sought to explore the effects of dotinurad therapy on other clinical measurements, including BP and vascular function, and the relationships between changes in the SUA level and those parameters in the planned sub-analyses. In this context, assuming that the correlation was approximately *r* = 0.40, the number of cases required would be 47 when calculated with *α* = 0.05 and 1 − β (power) = 0.80. Taking dropouts into account, the required sample size was set at 50.

SUA levels were evaluated at each visit (weeks 4, 8, 12, and 24) using a mixed model for repeated measures and compared with the baseline. According to several background characteristics stratified by gender, age, body mass index level, eGFR level, SUA level, histories of diabetes, dyslipidemia, and CVD, pre-specified subgroup analyses were also performed to explore the robustness of the primary endpoint. The prevalence of patients whose SUA levels reached ≤ 6 mg/dL was calculated, and the relationship between the SUA level at baseline and the prevalence of achieving an SUA level of ≤ 6 mg/dL at week 24 was examined using a logistic model as a post hoc analysis. To account for non-linear associations, a restricted cubic spline with three knots was included in the model. Other secondary endpoints were evaluated generally in the same manner as the primary endpoint. For variables for which the measured values were non-normally distributed, the proportional changes in the pre- and post-geometric means were calculated. Unless otherwise stated, the significance level was set at 5% two sided. No adjustments for multiple comparisons were considered. All statistical analyses were performed using R software version 4.2.0 (R Foundation for Statistical Computing, Vienna, Austria).

## Results

### Patient characteristics

**Fig. 1:**
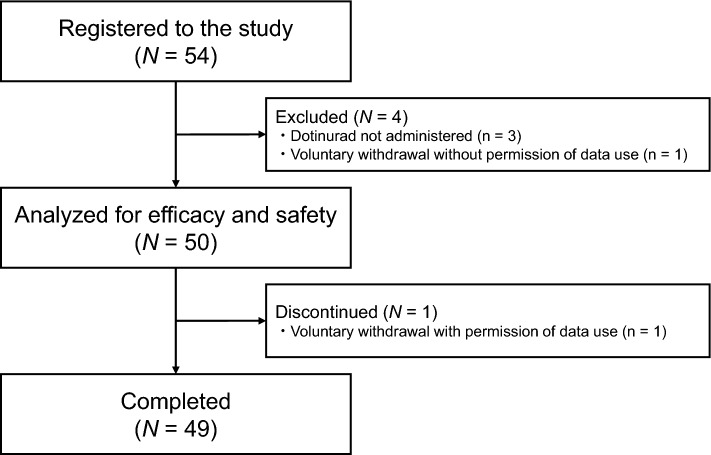
Flowchart of the study participants

Of the 54 patients screened and registered, 50 met the eligibility criteria and were included in the efficacy and safety analysis (Fig. 1), and 49 patients completed the study. The demographics and baseline characteristics of patients are shown in Table 1. The mean patient age was 70.5 ± 11.0 years, in which 76.0% were men, and the mean SUA level was 8.5 ± 1.2 mg/dL at registration. A total of 37 (74.0%) patients were naïve for urate-lowering agents, and the remaining had been taking urate-lowering agents by at least 27 days before the initiation of dotinurad therapy.

**Table 1:** Baseline demographics and characteristics of the participants

Variables	Analyzed subjects (*N* = 50)
Age, years	70.5 ± 11.0
Male	38 (76.0)
Body mass index, kg/m^2^	24.7 ± 4.0
Systolic blood pressure, mmHg	131.2 ± 17.0
Diastolic blood pressure, mmHg	75.0 ± 11.3
Serum uric acid, mg/dL	8.5 ± 1.2
Active gouty arthritis	0 (0.0)
Hypertension	50 (100.0)
Dyslipidemia	37 (74.0)
Diabetes	19 (38.0)
Ischemic heart disease	16 (32.0)
Heart failure	10 (20.0)
Stroke	0 (0.0)
Medications	
ACE inhibitor or ARB	44 (88.0)
Calcium channel blocker	25 (50.0)
β-blocker	31 (62.0)
Diuretic	19 (38.0)
SGLT2 inhibitor	13 (26.0)
Statin	34 (68.0)
Ezetimibe	9 (18.0)
Anti-platelet	16 (32.0)
Previous* use of urate-lowering agent	13 (26.0)

Data are expressed as mean ± standard deviation or number (percentage)

*At least 27 days before the initiation of dotinurad

ACE: angiotensin-converting enzyme; ARB: angiotensin receptor blocker; SGLT2: sodium-glucose cotransporter 2

The prevalence of the doses of dotinurad at each visit is shown in Additional file 2: Fig. S1). At week 24, 38 (76.0%) patients received 2 mg of dotinurad once daily.

### Effects on SUA levels

**Fig. 2:**
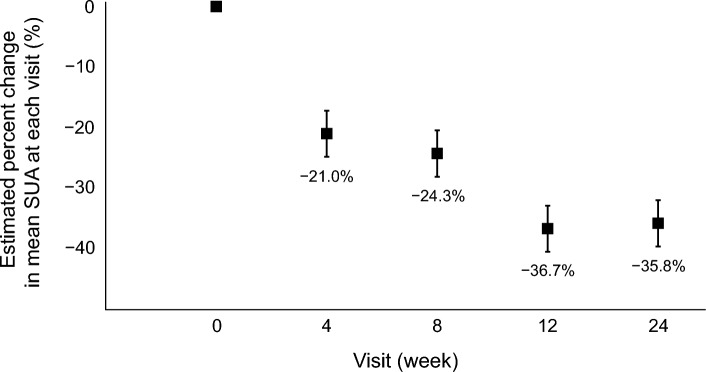
Estimated percent change in the mean SUA level at week 24. Bars: 95% confidence interval. SUA: serum uric acid

**Fig. 3:**
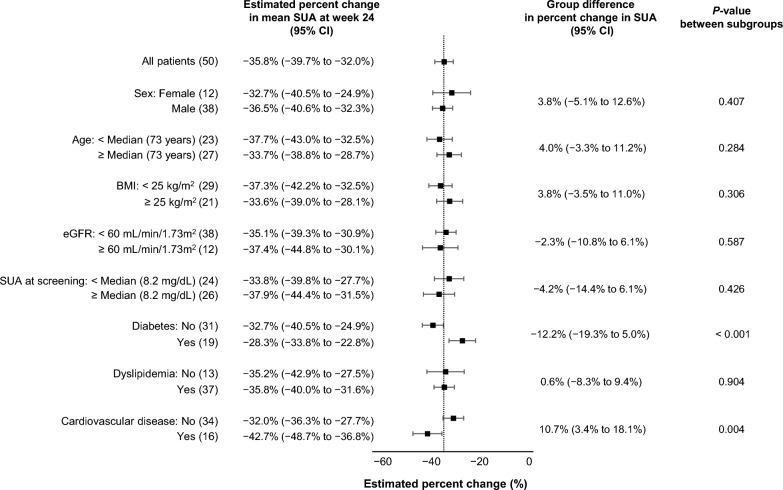
Subgroup analyses for primary endpoint. BMI: body mass index; CI: confidence interval; eGFR: estimated glomerular filtration rate; SUA: serum uric acid

The raw SUA levels at baseline and weeks 4, 8, 12, and 24 are provided in Additional file 3: Table S2. The estimated mean values and 95% CIs of the SUA levels measured at each time point and changes from baseline using a mixed-effect model are shown in Table 2. As illustrated in Fig. 2, the percentage change in the SUA level from baseline was − 21.0% (95% CI − 24.8% to − 17.2%) at week 4, − 24.3% (95% CI − 28.1% to − 20.5%) at week 8, − 36.7% (95% CI − 40.6% to − 32.9%) at week 12, − 35.8% (95% CI − 39.7% to − 32.0%) at week 24 (primary endpoint of the study). In the pre-specified subgroup analyses for the primary endpoint, the treatment effect was almost consistent, except for subgroups by a history of diabetes or CVD (Fig. 3).

**Table 2:** Estimated changes in SUA concentration over 24 weeks

Time point	Estimated mean value	95% CI	*P-*value
Baseline	8.25	7.96 to 8.55	
At week 4	6.48	6.18 to 6.78	
Absolute change from baseline	− 1.77	− 2.09 to − 1.45	< 0.001
Percentage change from baseline	− 21.0%	− 24.8% to − 17.2%	< 0.001
At week 8	6.16	5.86 to 6.46	
Absolute change from baseline	− 2.09	− 2.41 to − 1.77	< 0.001
Percentage change from baseline	− 24.3%	− 28.1% to − 20.5%	< 0.001
At week 12	5.16	4.86 to 5.46	
Absolute change from baseline	− 3.10	− 3.41 to − 2.78	< 0.001
Percentage change from baseline	− 36.7%	− 40.6% to − 32.9%	< 0.001
At week 24	5.24	4.94 to 5.54	
Absolute change from baseline	− 3.01	− 3.33 to − 2.69	< 0.001
Percentage change from baseline	− 35.8%	− 39.7% to − 32.0%	< 0.001

Unit of SUA is mg/dL

CI: confidence interval; SUA: serum uric acid

**Fig. 4:**
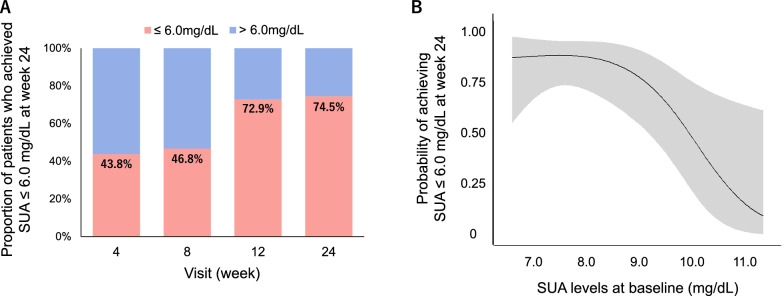
Proportion of patients who achieved SUA level of ≤ 6.0 mg/dL at week 24 (**A**) and achieving probability according to the SUA levels at baseline (**B**). Gray zone: 95% confidence interval. SUA: serum uric acid

The proportion of patients who achieved an SUA level of ≤ 6.0 mg/dL was 43.8% (21/48) at week 4, 46.8% (22/47) at week 8, 72.9% (35/48) at week 12, 74.5% (35/47) at week 24 (Fig. 4A). The probability of achieving an SUA level of ≤ 6.0 mg/dL, based on baseline SUA levels, is shown in Fig. 4B. The odds ratios achieving it for each 0.5 mg/dL lower SUA level at baseline are also shown in Additional file 4: Table S3.

### Other efficacy endpoints and safety

Systolic and diastolic BPs did not significantly change over 24 weeks (Additional file 5: Table S4). Meanwhile, the geometric mean of CAVI at baseline and week 24 were 9.29 (95% CI 8.83 to 9.78) and 8.92 (95% CI 8.47 to 9.40), respectively, and the proportional change in CAVI from baseline to week 24 was 0.96 (95% CI 0.92 to 1.00 *P* = 0.044). Dotinurad therapy did not affect the levels of the following biomarkers: eGFR, CRP, GDF-15, NT-proBNP, hs-TnT, and UACR, except for IL-6. The d-ROMs concentration at week 24 was significantly lower than that at baseline (a proportional change in the geometric mean of d-ROMs concentration, 0.96 [95% CI 0.92 to 1.00], *P* = 0.044), and the Fib-4 index at week 24 tended to be lower than that at baseline (a proportional change in the geometric mean of Fib-4 index, 0.96 [95% CI 0.91 to 1.00], *P* = 0.061) (Additional file 5: Table S4).

During the study, site investigators reported four adverse events, namely, mild elbow pain, stomach pain, eruption, and lateral abdominal pain. No new onset of a gout attack or nephrolithiasis was reported.

## Discussion

The major findings of this exploratory study (DIANA) of patients with hyperuricemia and treated hypertension were as follows: (i) the 24-week dotinurad therapy effectively reduced the SUA levels without excess harm of drug reaction, (ii) the treatment improved the arterial stiffness marker as assessed by CAVI, and (iii) the treatment significantly attenuated the oxidative stress biomarker as assessed by d-ROMs concentration. To the best of our knowledge, this is the first study showing clinical evidence of the adequate urate-lowering effect of dotinurad on patients with hyperuricemia and treated hypertension. Our findings also suggest that dotinurad has the potential to provide some off-target effects, which may favorably affect cardiovascular and metabolic health status.

Dotinurad is a newer urate-lowering agent that selectively inhibits URAT1 located at the renal proximal tubules and increases uricosuria excretion [6]. URAT1 is exclusively responsible for urate reabsorption in the kidney [16], and previous studies have demonstrated that the function is activated by increased insulin resistance and impaired cardiometabolic health, including hypertension and diabetes, resulting in high SUA levels [7, 17]. By contrast, the urate-lowering efficacy by conventional urate-lowering agents, such as benzbromarone or febuxostat that potentially inhibit ABCG2, an extrarenal (intestinal) urate excretion transporter [18], would be theoretically diminished under such conditions. These suggest that selective inhibition of URAT1 is a reasonable therapy to reduce effectively the SUA levels in such patient populations with renal urate underexcretion [19]. Therefore, we sought to assess the effects of dotinurad therapy primarily on the SUA levels in patients with hyperuricemia complicated with hypertension and found that the therapy substantially reduced the SUA levels over 24 weeks, as observed in previous phase 2 and 3 clinical studies with dotinurad in general patient populations with hyperuricemia [20, 21].

A high SUA level is a residual risk factor or marker of CVD [22]. However, whether urate-lowering therapy can reduce CVD risk is still inconclusive [23–25]. Several observational and case–control studies have demonstrated that treatment with conventional XO inhibitor allopurinol was associated with reduced CVD risk [26, 27]. Meanwhile, recent randomized clinical trials with some XO inhibitors, such as allopurinol and febuxostat, showed they have no obvious cardiovascular benefits [28–30]. Intriguingly, several cohort studies have consistently demonstrated that the conventional uricosuric agents probenecid and benzbromarone, compared with allopurinol, were associated with reduced CVD risk [31, 32]. Thus, potential differences may appear in the prevention of CVD between the two classes of urate-lowering agents, and uricosuric agents may favorably affect cardiometabolic properties. Nevertheless, clinical evidence on the cardiovascular and metabolic effects of uricosuric agents, including dotinurad, is currently lacking. In addition, whether dotinurad has better effects on those properties than allopurinol or febuxostat is still unknown.

The SUA level is positively associated with increased arterial stiffness in the general population, including individuals with hypertension [33, 34]. However, data are conflicting on the effect of urate-lowering medications on arterial stiffness markers. A meta-analysis showed that allopurinol therapy failed to improve arterial stiffness as measured by pulse wave velocity (PWV) [35]. Shiina et al. recently reported that a 24-month febuxostat therapy improved arterial stiffness markers by combining PWV and CAVI in patients with asymptomatic hyperuricemia [12]. Meanwhile, data about the effects of uricosuric agents on arterial stiffness are currently limited, and the difference in the effects between the two classes of urate-lowering agents is also uncertain.

In this study, the values of an arterial stiffness marker (CAVI) and an oxidative stress marker (d-ROMs) after 24 weeks of dotinurad therapy were significantly lower than their baseline. In addition, the Fib-4 index (a liver fibrosis maker) at week 24 tended to be lower than that at baseline. The precise mechanisms responsible for those findings are still unclear. Taufiq et al. showed that dotinurad suppressed monosodium urate-induced activation of nucleotide-binding oligomerization domain-like receptor family pyrin domain-containing protein 3 inflammasomes in mouse macrophages [36]. A recent experimental study also revealed that dotinurad ameliorated insulin resistance and hepatic steatosis through the suppression of reactive oxygen species (ROS) production and brown adipose tissue whitening in high-fat diet-induced obese mice [37]. At least, no clear relationships of those changes with urate-lowering were found in this study (Additional file 6: Table S5), and the beneficial vascular effects might have been partly caused by selective and direct URAT1 inhibition by dotinurad at the urate-entry site on vascular walls and resultant attenuation of ROS production [8, 9]. In particular, arterial stiffening is a key driver of hypertension and could be a therapeutic target in its care [38, 39]. Considering the pathophysiological roles of those markers in the development and progression of cardiometabolic diseases, dotinurad may have the potential to cause off-target effects, which favorably influence vascular properties and cardiovascular and metabolic health status.

In this study, other clinical parameters tested, including BP and renal function, did not change over 24 weeks. Our results regarding the effect of BP are consistent with those of most previous studies investigating the effect of urate-lowering medications [40–42], but not all [43]. This inconsistency might be partly caused by the differences in the agents used, study design, and population. Moreover, a meta-analysis showed low-certainty evidence that urate-lowering medication, mainly XO inhibitors, reduces BP in patients with hyperuricemia [44]. Regarding the effect of renal function, some urate-lowering medications mitigated kidney function decline in patients with hyperuricemia and chronic kidney disease [45, 46]. However, the effect of urate-lowering medications on renal function may differ according to renal function [47]. Importantly, no clinical data on the detailed effect of dotinurad on renal function are currently available. A study investigating the clinical efficacy and safety of dotinurad in patients with hyperuricemia and diabetic kidney disease is now ongoing [48]. Thus, further research is expected to uncover the entire off-target effects of dotinurad on non-urate clinical status, including BP and renal function.

### Study limitations

This study has some limitations. First, this is a non-randomized, single-arm, open-label, exploratory study with relatively small sample size and short-term intervention. This may limit the interpretation of whether dotinurad specifically influenced the endpoints. Therefore, our findings should be further verified in randomized studies with an appropriate sample size and an appropriate control group. Second, dose adjustment (up-titration) of dotinurad was not uniformly performed in all participants. The up-titration of dotinurad was based on the site investigators’ judgment in a clinically pragmatic fashion. Although the maintenance dose of 2 mg daily was used at week 24 in 76% of the patients, no one received its maximum dose of 4 mg daily during the study interval. Therefore, determining the dose-dependence property of dotinurad on the study endpoints is impossible. Third, the clinical subtype of hyperuricemia based on its etiology, such as overproduction or underexcretion of urate, was not identified in the study participants. Fourth, this study focused on clinically stable Japanese patients with hyperuricemia and treated hypertension; thus, the generalizability of our findings to patient populations with other comorbidities and clinical situations is still uncertain. Importantly, the indication to treat asymptomatic hyperuricemia is still highly debated, as many countries do not recommend treating it, unlike Japan. Given that dotinurad is currently approved only in Japan, further studies are needed to determine its clinical efficacy in other ethnicities or regions and symptomatic hyperuricemia. Finally, we had no measurement of carotid-femoral PWV, which has been widely recognized as a gold standard method to assess central arterial stiffness non-invasively [49, 50].

## Conclusion

In this exploratory DIANA study, 24 weeks of dotinurad therapy in patients with hyperuricemia and treated hypertension showed a clinically meaningful urate-lowering effect. In addition, the treatment may favorably affect arterial stiffness and oxidative stress markers. Further research is expected to verify our findings and elucidate the entire off-target effects of dotinurad on non-urate cardiovascular and metabolic health status.

## Supplementary Information

**Additional file 1: Table S1.** Eligibility criteria for the DIANA study.

**Additional file 2: Fig. S1.** Doses of dotinurad at each visit.

**Additional file 3: Table S2.** SUA raw data at baseline and at weeks 4, 8, 12, and 24.

**Additional file 4: Table S3.** Odds ratios for achieving an SUA level of ≤ 6.0 mg/dL according to baseline SUA levels.

**Additional file 5: Table S4.** Estimated changes in blood pressures and laboratory biomarkers over 24 weeks.

**Additional file 6: Table S5.** Associations between changes in SUA and parameters of interest at week 24.

**Additional file 7: Text S1.** DIANA Study Organization and Investigators.

## Data Availability

The datasets generated during and/or analyzed during the current study are not publicly available due to ethical restrictions but are available from the corresponding author upon reasonable request.

## Abbreviations

BP: Blood pressure
CAVI: Cardio-ankle vascular index
CI: Confidence interval
CRP: C-reactive protein
CVD: Cardiovascular disease
d-ROMs: Derivatives of reactive oxygen metabolites
eGFR: Estimated glomerular filtration rate
GDF-15: Growth differentiation factor 15
hs-TnT: High-sensitivity troponin T
IL-6: Interleukin-6
NT-proBNP: N-terminal pro-brain natriuretic peptide
PWV: Pulse wave velocity
SUA: Serum uric acid
UACR: Urinary albumin creatinine ratio
URAT1: Urate transporter 1
