# Supplementary material for: Clinical effects of a selective urate reabsorption inhibitor dotinurad in patients with hyperuricemia and treated hypertension: a multicenter, prospective, exploratory study (DIANA)
Source: Eur J Med Res. 2023 Jul 17;28:238. doi: 10.1186/s40001-023-01208-1 (PMC10351195; doi:10.1186/s40001-023-01208-1)
Supplement: Supplementary file 1 — Additional file 1: Table S1. Eligibility criteria for the DIANA study. [file 40001_2023_1208_MOESM1_ESM.docx]

**Additional file 1: Table S1** Eligibility criteria for the DIANA study

| **Inclusion** | **Exclusion** |
| --- | --- |
| 1. Patients aged ≥20 years at the time when consent was obtained (regardless of sex). 2. Patients with hyperuricemia and a serum uric acid level of >7.0 mg/dL. 3. Patients with hypertension, who meet the diagnostic criteria based on the guidelines* for the management of hypertension 2019 by the Japanese Society of Hypertension, without change in the pharmacological and/or non-pharmacological treatment for hypertension within 4 weeks before eligibility assessment. 4. Patients who provided written consent to participate in the study. | 1. Patients with active gouty arthritis. 2. Patients who are currently suffering from urolithiasis. 3. Patients with known secondary hyperuricemia who are suffering from any of the following diseases: Lesch–Nyhan syndrome, phosphoribosyl pyrophosphate synthetase superactivity, congenital myogenic hyperuricemia, hematopoietic organ tumor (acute leukemia, malignant lymphoma, myeloproliferative disorder, and myelodysplastic syndrome), solid tumor (breast cancer, seminoma, sarcoma, Wilms tumor, and small cell lung cancer), non-neoplastic disease (psoriasis vulgaris, secondary polycythemia, and hemolytic anemia), tumor lysis syndrome, rhabdomyolysis, hypothyroidism, polycystic kidney, lead poisoning/lead nephropathy, Down’s syndrome, familial juvenile gouty nephropathy, hyperlactacidemia, or glycogen storage disease type I. 4. Patients with hypertensive emergency and urgency. 5. Patients with active malignancy. 6. Patients with severe hepatic dysfunction. 7. Patients with severe renal dysfunction with oliguria or anuria. 8. Patients who are pregnant, suspected to be pregnant, or breastfeeding. 9. Patients with a history of hypersensitivity to the ingredients contained in dotinurad. 10. Patients considered by the study investigators not suitable for participation in the study. |

* Umemura S, Arima H, Arima S, et al. The Japanese Society of Hypertension Guidelines for the Management of Hypertension (JSH 2019). Hypertens Res. 2019;42(9):1235-1481.
