# Supplementary figures and images for: Clinical effects of a selective urate reabsorption inhibitor dotinurad in patients with hyperuricemia and treated hypertension: a multicenter, prospective, exploratory study (DIANA)
Source: Eur J Med Res. 2023 Jul 17;28:238. doi: 10.1186/s40001-023-01208-1 (PMC10351195; doi:10.1186/s40001-023-01208-1)

**Additional file 2: Fig. S1** Doses of dotinurad at each visit


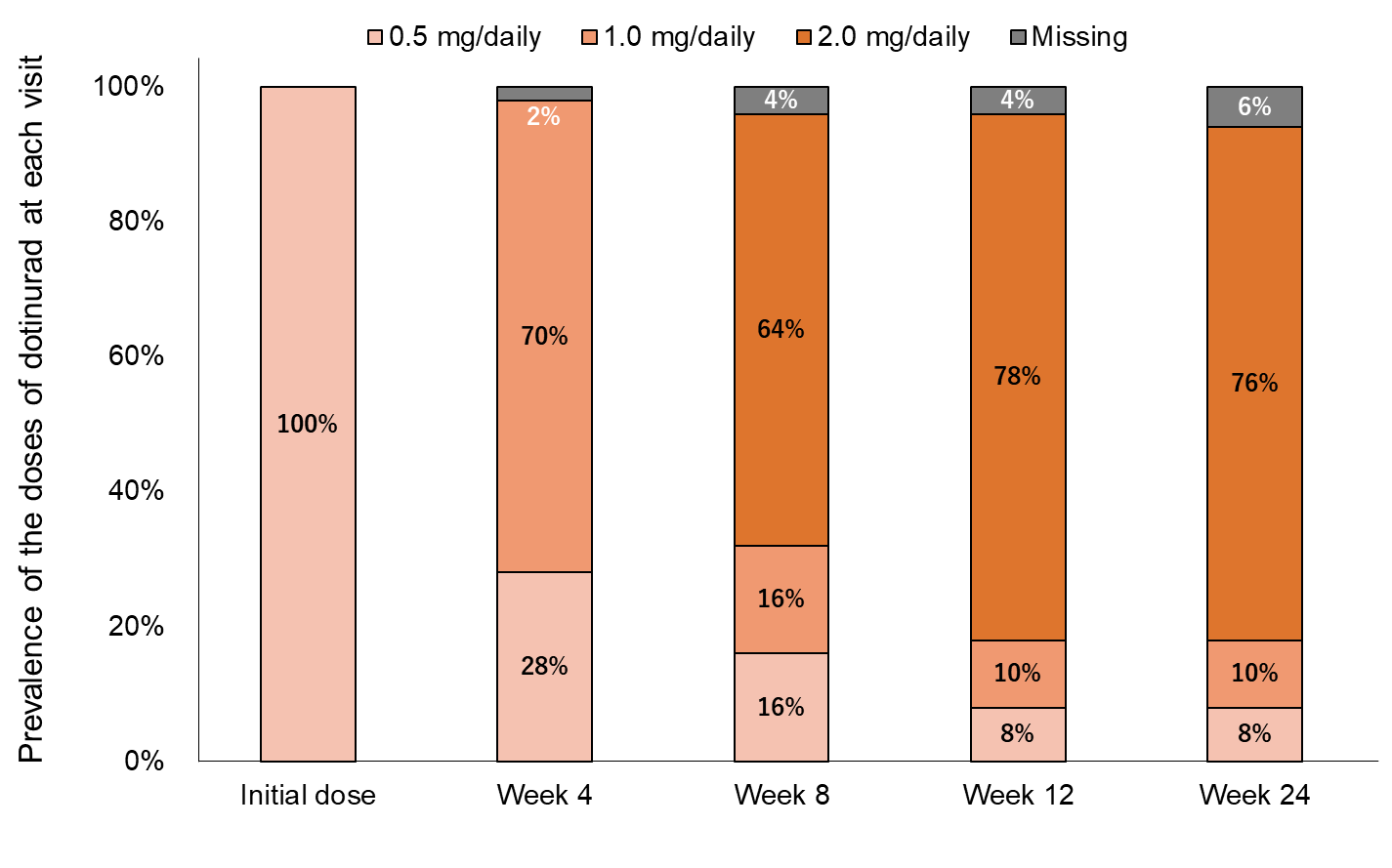

Supplement: Supplementary file 2 — Additional file 2: Fig. S1. Doses of dotinurad at each visit. [file 40001_2023_1208_MOESM2_ESM.docx]
