# Supplementary material for: Clinical effects of a selective urate reabsorption inhibitor dotinurad in patients with hyperuricemia and treated hypertension: a multicenter, prospective, exploratory study (DIANA)
Source: Eur J Med Res. 2023 Jul 17;28:238. doi: 10.1186/s40001-023-01208-1 (PMC10351195; doi:10.1186/s40001-023-01208-1)
Supplement: Supplementary file 3 — Additional file 3: Table S2. SUA raw data at baseline and at weeks 4, 8, 12, and 24. [file 40001_2023_1208_MOESM3_ESM.docx]

**Additional file 3: Table S2** SUA raw data at baseline and at weeks 4, 8, 12, and 24

| **Time point** | **Number** | **SUA, mg/dL** | **Missing** |
| --- | --- | --- | --- |
| Baseline | 50 | 8.4 ± 1.5 | 0 |
| At week 4 | 48 | 6.6 ± 1.5 | 2 |
| At week 8 | 47 | 6.3 ± 1.3 | 3 |
| At week 12 | 48 | 5.2 ± 1.5 | 2 |
| At week 24 | 47 | 5.4 ± 1.6 | 3 |

Data are expressed as mean ± standard deviation.

*SUA, serum uric acid.*
