# Supplementary material for: Clinical effects of a selective urate reabsorption inhibitor dotinurad in patients with hyperuricemia and treated hypertension: a multicenter, prospective, exploratory study (DIANA)
Source: Eur J Med Res. 2023 Jul 17;28:238. doi: 10.1186/s40001-023-01208-1 (PMC10351195; doi:10.1186/s40001-023-01208-1)
Supplement: Supplementary file 4 — Additional file 4: Table S3. Odds ratios for achieving an SUA level of ≤ 6.0 mg/dL according to baseline SUA levels. [file 40001_2023_1208_MOESM4_ESM.docx]

**Additional file 4: Table S3** Odds ratios for achieving an SUA level of ≤ 6.0 mg/dL according to baseline SUA levels

| **Baseline SUA level** | **Odd ratio** | **95% CI** | ***P-*value** |
| --- | --- | --- | --- |
| 7.0 vs. 7.5 (reference) | 0.959 | 0.486 to 1.894 | 0.905 |
| 7.5 vs. 8.0 (reference) | 1.070 | 0.625 to 1.832 | 0.805 |
| 8.0 vs. 8.5 (reference) | 1.285 | 0.911 to 1.813 | 0.153 |
| 8.5 vs. 9.0 (reference) | 1.558 | 1.135 to 2.140 | 0.006 |
| 9.0 vs. 9.5 (reference) | 1.817 | 1.155 to 2.859 | 0.010 |
| 9.5 vs. 10.0 (reference) | 2.035 | 1.126 to 3.679 | 0.019 |
| 10.0 vs. 10.5 (reference) | 2.188 | 1.099 to 4.357 | 0.026 |
| 10.5 vs. 11.0 (reference) | 2.261 | 1.087 to 4.703 | 0.029 |

Estimated by a logistic model including a restricted cubic spline with three knots.

*CI, confidence interval; SUA, serum uric acid.*
