# Supplementary material for: Clinical effects of a selective urate reabsorption inhibitor dotinurad in patients with hyperuricemia and treated hypertension: a multicenter, prospective, exploratory study (DIANA)
Source: Eur J Med Res. 2023 Jul 17;28:238. doi: 10.1186/s40001-023-01208-1 (PMC10351195; doi:10.1186/s40001-023-01208-1)
Supplement: Supplementary file 5 — Additional file 5: Table S4. Estimated changes in blood pressures and laboratory biomarkers over 24 weeks. [file 40001_2023_1208_MOESM5_ESM.docx]

**Additional file 5: Table S4** Estimated changes in blood pressures and laboratory biomarkers over 24 weeks

| **Variables** | **Time point** | **Estimated mean value** | **95% CI** | ***P-*value** |
| --- | --- | --- | --- | --- |
| Systolic BP, *mmHg* | Baseline | 131.2 | 126.5 to 135.9 |  |
|  | At week 4 | 128.3 | 123.6 to 133.0 |  |
|  | Absolute change from baseline | −3.0 | −7.0 to 0.9 | 0.133 |
|  | Percentage change from baseline | −2.0% | −5.0% to 1.0% | 0.196 |
|  | At week 8 | 132.3 | 127.6 to 137.0 |  |
|  | Absolute change from baseline | 1.0 | −3.0 to 4.9 | 0.632 |
|  | Percentage change from baseline | 1.4% | −1.6% to 4.4% | 0.369 |
|  | At week 12 | 128.1 | 123.4 to 132.9 |  |
|  | Absolute change from baseline | −3.1 | −7.1 to 0.8 | 0.119 |
|  | Percentage change from baseline | −1.6% | −4.7% to 1.4% | 0.293 |
|  | At week 24 | 129.0 | 124.3 to 133.8 |  |
|  | Absolute change from baseline | −2.3 | −6.2 to 1.7 | 0.261 |
|  | Percentage change from baseline | −1.1% | −4.2% to 2.0% | 0.477 |
| Diastolic BP, *mmHg* | Baseline | 75.0 | 71.9 to 78.2 |  |
|  | At week 4 | 75.4 | 72.2 to 78.6 |  |
|  | Absolute change from baseline | 0.5 | −2.3 to 3.3 | 0.750 |
|  | Percentage change from baseline | 1.2% | −2.8% to 5.2% | 0.561 |
|  | At week 8 | 77.4 | 74.2 to 80.6 |  |
|  | Absolute change from baseline | 2.2 | −0.6 to 5.0 | 0.118 |
|  | Percentage change from baseline | 4.3% | 0.3% to 8.3% | 0.036 |
|  | At week 12 | 74.8 | 71.6 to 77.9 |  |
|  | Absolute change from baseline | −0.4 | −3.2 to 2.4 | 0.804 |
|  | Percentage change from baseline | 0.6% | −3.4% to 4.6% | 0.755 |
|  | At week 24 | 75.9 | 72.7 to 79.1 |  |
|  | Absolute change from baseline | 0.8 | −2.1 to 3.6 | 0.600 |
|  | Percentage change from baseline | 2.3% | −1.7% to 6.3% | 0.263 |
| eGFR, *mL/min/1.73 m^2^** | Baseline | 47.8 | 43.6 to 52.3 |  |
|  | At week 12 | 47.2 | 43.1 to 51.6 |  |
|  | Proportional change from baseline* | 0.99 | 0.95 to 1.02 | 0.425 |
|  | At week 24 | 46.9 | 42.8 to 51.4 |  |
|  | Proportional change from baseline* | 0.98 | 0.95 to 1.01 | 0.265 |
| Fib-4 index* | Baseline | 1.92 | 1.66 to 2.22 |  |
|  | At week 12 | 1.93 | 1.67 to 2.24 |  |
|  | Proportional change from baseline | 1.01 | 0.96 to 1.06 | 0.678 |
|  | At week 24 | 1.83 | 1.58 to 2.12 |  |
|  | Proportional change from baseline | 0.96 | 0.91 to 1.00 | 0.061 |
| CRP, mg/dL* | Baseline | 0.07 | 0.05 to 0.09 |  |
|  | At week 24 | 0.06 | 0.05 to 0.09 |  |
|  | Proportional change from baseline | 0.96 | 0.78 to 1.19 | 0.727 |
| IL-6, pg/mL* | Baseline | 3.33 | 2.82 to 3.93 |  |
|  | At week 24 | 3.82 | 3.22 to 4.52 |  |
|  | Proportional change from baseline | 1.14 | 1.00 to 1.31 | 0.047 |
| GDF-15, pg/mL* | Baseline | 2136.1 | 1789.3 to 2550.0 |  |
|  | At week 24 | 2194.4 | 1836.6 to 2622.0 |  |
|  | Proportional change from baseline | 1.03 | 0.95 to 1.11 | 0.479 |
| NT-proBNP, pg/mL* | Baseline | 196.8 | 132.0 to 293.6 |  |
|  | At week 24 | 173.8 | 116.3 to 259.9 |  |
|  | Proportional change from baseline | 0.88 | 0.73 to 1.06 | 0.190 |
| hs-TnT, pg/mL* | Baseline | 15.5 | 12.3 to 19.4 |  |
|  | At week 24 | 17.3 | 13.7 to 21.9 |  |
|  | Proportional change from baseline | 1.12 | 0.91 to 1.38 | 0.297 |
| d-ROMs, U.CARR* | Baseline | 343.5 | 319.1 to 369.7 |  |
|  | At week 24 | 329.8 | 306.1 to 355.4 |  |
|  | Proportional change from baseline | 0.96 | 0.92 to 1.00 | 0.044 |
| UACR, *mg/g.cr** | Baseline | 32.7 | 19.2 to 55.7 |  |
|  | At week 24 | 27.2 | 15.9 to 46.4 |  |
|  | Proportional change from baseline | 0.83 | 0.62 to 1.12 | 0.230 |

Estimated in N = 50, excluding d-ROMs (N = 27) and UACR (N = 48).

*Corresponding data are expressed as geometric mean and 95% CI and the proportional changes from baseline estimated using a natural logarithmic scale.

*BP, blood pressure; CI, confidence interval; CRP, C-reactive protein; d-ROMs, derivatives of reactive oxygen metabolites; eGFR, estimated glomerular filtration rate; GDF-15, growth differentiation factor 15; hs-TnT, high-sensitivity troponin T; IL-6, interleukin-6; NT-proBNP, N-terminal pro-brain natriuretic peptide; UACR, urinary albumin creatinine ratio.*
