# Supplementary material for: Clinical effects of a selective urate reabsorption inhibitor dotinurad in patients with hyperuricemia and treated hypertension: a multicenter, prospective, exploratory study (DIANA)
Source: Eur J Med Res. 2023 Jul 17;28:238. doi: 10.1186/s40001-023-01208-1 (PMC10351195; doi:10.1186/s40001-023-01208-1)
Supplement: Supplementary file 6 — Additional file 6: Table S5. Associations between changes in SUA and parameters of interest at week 24. [file 40001_2023_1208_MOESM6_ESM.docx]

**Additional file 6: Table S5** Associations between changes in SUA and parameters of interest at week 24

| **Variables** | ***r*** | **95% CI** | ***P-*value** |
| --- | --- | --- | --- |
| CAVI | −0.094 | −0.371 to 0.198 | 0.530 |
| Fib-4 index | 0.005 | −0.283 to 0.292 | 0.974 |
| d-ROMs | 0.119 | −0.299 to 0.499 | 0.579 |

Calculated by Spearman’s rank correlation coefficients.

*CAVI, cardio-ankle vascular index; CI, confidence interval; d-ROMs, derivatives of reactive oxygen metabolites; SUA, serum uric acid.*
