# Supplementary material for: Clinical effects of a selective urate reabsorption inhibitor dotinurad in patients with hyperuricemia and treated hypertension: a multicenter, prospective, exploratory study (DIANA)
Source: Eur J Med Res. 2023 Jul 17;28:238. doi: 10.1186/s40001-023-01208-1 (PMC10351195; doi:10.1186/s40001-023-01208-1)
Supplement: Supplementary file 7 — Additional file 7: Text S1. DIANA Study Organization and Investigators. [file 40001_2023_1208_MOESM7_ESM.docx]

**Additional file 7: Text S1** DIANA Study Organization and Investigators

**Principal Investigator:** Dr. Koichi Node (Saga University, Saga, Japan).

**Site Investigators:**

*Saga University (Saga, Japan)*

Drs. Machiko Asaka, Hiroshi Hongo, Kohei Kamishita, Tetsuya Kaneko, Kohei Kaneta, Masahiro Natsuaki, Koichi Node, Aya Shiraki, Shinjo Sonoda, Motoko Tago, Atsushi Tanaka, Ayumu Yajima, Kensuke Yokoi, and Goro Yoshioka.

*Dokkyo Medical University Saitama Medical Center (Saitama, Japan)*

Drs. Itaru Hisauchi, Tetsuya Ishikawa, and Isao Taguchi.

*Fukuoka Saiseikai Futsukaichi Hospital (Chikushino, Japan)*

Drs. Toshiaki Kadokami, Ryo Nakamura, Junichiro Nishi, and Ken Onizuka.

*Tokushima University Hospital (Tokushima, Japan)*

Drs. Takayuki Ise, Muneyuki Kadota, Yutaka Kawabata, Kenya Kusunose, Kazuhisa Matsumoto, Tomomi Matsuura, Yuichiro Okushi, Masataka Sata, Hiromitsu Seno, Takeshi Soeki, Kumiko Suto, Tomonori Takahashi, Takeshi Tobiume, Tetsuzo Wakatsuki, Shusuke Yagi, Hirotsugu Yamada, and Koji Yamaguchi.

*Fukushima Medical University School of Medicine (Fukushima, Japan)*

Drs. Yuki Hotta, Mariko Iwasaki, Junichiro Kazama, Yu Saito, Masahiro Sato, Michio Shimabukuro, Yoshinori Takiguchi, Hayato Tanabe, Kiriko Watanabe, and Mizuki Yamaguchi.

**Statistics:** Ms. Hisako Yoshida (Organization for Clinical Medicine Promotion, Tokyo, Japan).

**Data Management and Monitoring:** Dr. Takuya Kishi (Organization for Clinical Medicine Promotion, Tokyo, Japan).

**Study Secretariat:** Dr. Atsushi Tanaka (Saga University, Saga, Japan), Ms. Sachiko Tomita (Saga University, Saga, Japan), Ms. Mikiko Kagiyama (Saga University, Saga, Japan); Ms. Keiko Onodera (Organization for Clinical Medicine Promotion, Tokyo, Japan.
